# Comparative Analysis of Oral Clonidine Versus Oral Gabapentin on Subarachnoid Block Duration and Postoperative Analgesic Needs

**DOI:** 10.7759/cureus.68834

**Published:** 2024-09-06

**Authors:** Aparna Bagle, Ishan G Garud

**Affiliations:** 1 Anesthesiology, Dr. D.Y. Patil Medical College, Hospital and Research Centre, Dr. D.Y. Patil Vidyapeeth (Deemed to be University) Pimpri, Pune, IND

**Keywords:** oral clonidine, oral gabapentin, post operative analgesia, post-operative nausa and vomitting(ponv), sub arachnoid block

## Abstract

Introduction: Pain, an inherently subjective experience, plays a pivotal role in the body’s defence mechanism by signalling tissue damage or potential harm. Thus, optimal postoperative pain management is a cornerstone of modern surgical practice, essential for improving recovery outcomes and overall patient well-being.

Methods: In this study, a total of 60 patients were randomly assigned to two groups of 30 each: Group C (clonidine) and Group G (gabapentin). Group C received oral clonidine 100 mcg and Group G received oral gabapentin 600 mg one hour prior to the subarachnoid block. Duration of sensory and motor block, hemodynamic parameters, Visual Analog Scale (VAS) scores, time to rescue analgesia and any side effect of both drugs were assessed in both groups.

Results: The duration of motor and sensory blockade, as well as intraoperative hemodynamics and respiratory rates, were comparable between the two groups. Group G exhibited significantly lower VAS scores from 150 minutes postoperatively up to 12 hours (p < 0.001). Additionally, Group G experienced longer duration of postoperative analgesia (16.8±3.9 hours) compared to Group C (9.27±1.7 hours). About 26.6% of the patients in Group C and 6.7% of the patients in Group G presented with postoperative nausea and vomiting (PONV).

Conclusion: Oral gabapentin at a dosage of 600 mg demonstrates superior efficacy as a premedication compared to oral clonidine 100 mcg for patients undergoing lower abdominal and lower limb surgeries under spinal anesthesia. Group G demonstrated extended postoperative analgesia, lower VAS scores, and a reduced incidence of PONV, indicating its superiority over clonidine as an analgesic adjunct.

## Introduction

The pursuit of optimal pain management in modern anesthesia practice is a paramount objective, aimed at providing adequate analgesia while minimizing adverse effects and facilitating a smooth postoperative recovery. In the postoperative setting, uncontrolled pain can hinder recovery, extend hospitalization, and increase the risk of developing chronic pain syndromes. Effective postoperative analgesia is paramount in mitigating these risks. By adequately managing pain, patients can participate more actively in rehabilitation, including early mobilization and respiratory exercises, which are critical for preventing complications. Furthermore, reducing pain can lower the physiological stress response, promote healing, and enhance patient satisfaction. Subarachnoid block, also known as spinal anesthesia, has emerged as a widely utilized technique for lower abdominal, pelvic, and lower limb surgeries due to its reliable sensory and motor blockade, minimized hemodynamic instability, and reduced postoperative thromboembolic complications [1]. However, one of the significant limitations of the subarachnoid block is its relatively short duration of action, necessitating the administration of supplemental analgesics in the postoperative period to maintain adequate pain control [2].

To mitigate this limitation and enhance postoperative pain management, researchers have investigated the application of various adjuvants(oral, intravenous, intrathecal) in conjunction with local anesthetics for subarachnoid block. Notably, clonidine and gabapentin have attracted considerable interest for their potential to extend the duration of subarachnoid block and decrease postoperative analgesic demands.

This study focused on evaluating how oral clonidine and gabapentin tablets affect the extent of sensory and motor subarachnoid block, as well as postoperative analgesia. The secondary objectives included comparing the timing of the first rescue analgesic postoperatively, postoperative Visual Analog Scale (VAS) scores, and the total analgesic doses required within the first 24 hours after surgery.

## Materials and methods

After receiving the approval of the Institutional Ethics Committee of Dr. D.Y. Patil Medical College, Hospital and Research Centre, Dr. D.Y. Patil Vidyapeeth (Deemed to be University) Pimpri, Pune (IESC/PGS/2020/150) and Clinical Trials Registry - India (CTRI) clearance (CTRI/2021/11/038110), this prospective, randomized and comparative study was started.

The inclusion criteria encompassed male and female patients aged 18 to 65 years, classified as American Society of Anesthesiologists (ASA) grade I or II [3], scheduled for infraumbilical surgeries under subarachnoid block, and with informed consent obtained. Exclusion criteria included patients with ASA status III or higher [3], those with significant neurological, cardiac, respiratory, metabolic, renal, or hepatic disorders, coagulation abnormalities, contraindications to spinal anesthesia, known allergies to the study drugs, and patients who declined participation in the study.

The sample size for this study was determined using OpenEpi version 3 software with a confidence interval of 95% and power of study 80%. The mean duration of analgesia in a previous study by Singh et al. [4] in the gabapentin group was 248.17±19.6 minutes and in the clonidine group was 217±12.3 minutes. Considering all the above, the sample size was calculated using OpenEpi version 3, total sample size came as 10, five in each group, but for better validation of the result we took a total sample size of 60, 30 in the gabapentin group (Group G) and 30 in the clonidine group (Group C) respectively.

A pre-anesthetic checkup was conducted the day before surgery, noting detailed history and complaints, and performing general and systemic examinations. Routine laboratory investigations were done. Patients were kept nil per os for solids six hours prior to surgery, and informed consent was obtained in the preoperative area. In the preoperative room, baseline vitals were recorded, IV access was established, and fluids were started. Patients in Group C were administered 100 mcg of oral clonidine, while those in Group G received 600 mg of oral gabapentin, with both medications given one hour prior to surgery. In the operating room, patients received 500 ml of Ringer's Lactate before spinal anaesthesia. A spinal needle of 26G (Quincke) was inserted in L3-L4 intervertebral space (prior skin infiltration with 2% lignocaine), the subarachnoid block was performed with 3 ml of 0.5% bupivacaine and the level achieved was to the T-6 level. Post-spinal anaesthesia, vital signs were recorded at specified intervals until the completion of the surgery. Patients were then shifted to the post anaesthesia care unit and hemodynamic monitoring was continued. The duration of motor blockade was defined as the time from subarachnoid block onset to the ability to lift both lower limbs. The duration of sensory blockade was from block onset to the return of tactile sensation at the L5 dermatome (in the greater toe of both the lower limbs). Postoperative pain was assessed using the VAS scale [5] every 30 minutes for the first four hours, then every four hours for the next 24 hours. The Ramsey sedation score (RSS) was assessed at two hours postoperatively. The duration of analgesia, measured as the time to first rescue analgesia, was documented. When VAS >3, 50 mg of IV tramadol was administered, and the number of rescue doses required in 24 hours was noted. Side effects such as dizziness, hypotension, sedation, drowsiness, nausea, vomiting, and shivering were recorded and treated accordingly.

Statistical analysis

At the end of the study, data were gathered and compiled for analysis. Statistical analysis was conducted using SPSS software (version 25.0; IBM Corp., Armonk, NY, USA). Quantitative and qualitative variables were analyzed with the unpaired t-test and chi-square test respectively. A p-value below 0.05 was considered statistically significant.

## Results

**Figure 1:**
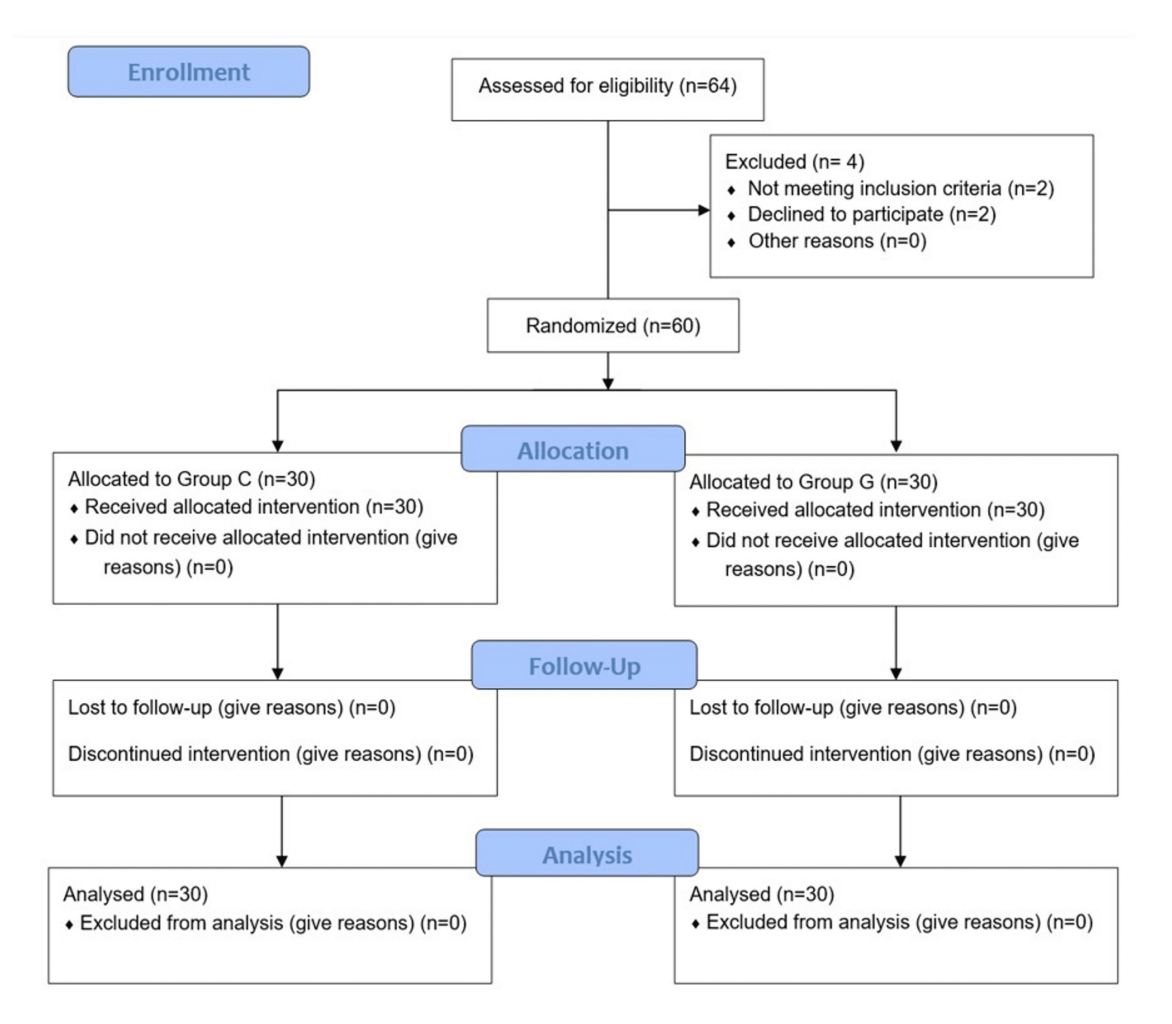
CONSORT Chart CONSORT: Consolidated Standards of Reporting Trials.

In this randomized comparative study, 64 patients were initially assessed, with four excluded due to not meeting the inclusion criteria and not willing to participate in the study . The remaining 60 patients were randomly assigned to two groups: Group C and Group G, each consisting of 30 participants. All patients completed the study and were included in the analysis as seen in Figure 1.

Demographic parameters

The study was completed by a total of 30 patients in each group. Both groups showed similarities in terms of age and gender distribution. Group C had a mean age of 42.23 years with 53.3% males and 46.7% females. Group G had a mean age of 39.7 years with 70% males and 30% females. The two study groups were comparable in terms of demographic characteristics, including age, gender, BMI, and types of surgeries, with no statistically significant differences as seen in Table 1.

**Table 1:** Comparison of Demographic Parameters Age and BMI presented as Mean±SD. Gender and Type of surgeries presented as n(%).

Parameters	Group C (n=30)	Group G (n=30)	p-value
Age (years)	43.23±10.4	39.73±10.6	0.205
Gender	Males- 16 (53.3%) Females- 14 (46.7%)	Male- 21 (70%) Females- 9 (30%)	0.184
BMI (in kg/m^2^)	20.90±3.39	21.12±2.96	0.7
Types of surgeries	-	-	-
Hydrocele repair	4(13.3%)	4(13.3%)	Not Applicable
Open Appendicectomy	7(23.3%)	10 (33.3%)	Not Applicable
Varicose veins Repair	11 (36.7%)	8(26.6%)	Not Applicable
Inguinal hernia	8(26.6%)	8(26.6%)	Not Applicable

Duration of sensory and motor block

Table 2 shows a comparison of the time period of motor and sensory blockade between the two groups. The average time period of both motor and sensory blockade was nearly identical between the groups and the difference in the groups was not significant when the independent ‘t’ test was applied (p>0.05).

**Table 2:** Duration of motor and sensory blockade among two study groups Data presented as Mean±SD. A p-value less than 0.05 indicates statistically significant differences between groups. However, the data provided above does not show such significant differences.

Duration of motor blockade	GROUP C	GROUP G	P value
Duration of motor blockade in minutes	198.55±18.68	196.60±16.17	0.6
Duration of sensory blockade in minutes	292.86±31.42	296.53±14.76	0.5

VAS scores

Group G consistently reports lower VAS scores compared to Group C as seen in Table 3. The difference in VAS scores between the groups increases over time. At 24 hours, Group C reports nearly twice the pain level of Group G. The difference in the groups was statistically significant at all mentioned time points (p<0.05).

**Table 3:** Visual Analog Scale (VAS) in the two groups at different intervals of time Data is presented as Mean±SD. (*) denote p-values with statistically significant differences between the two groups, where p-values are less than 0.05.

Time (h)	GROUP C	GROUP G	P value
Post operative 30 minutes	0	0	-
Post operative 60 minutes	0	0	-
Post operative 90 minutes	0	0	-
Post operative 120 minutes	0	0	-
Post operative 150 minutes	1.63±0.76	0.3±0.46	<0.001*
Post operative 180 minutes	2.03±0.76	0.8±0.61	<0.001*
Post operative 210 minutes	2.07±0.74	0.73±0.64	<0.001*
Post operative 240 minutes	2.07±0.74	0.7±0.65	<0.001*
Post operative 6 hours	3.03±0.71	1.2±0.66	<0.001*
Post operative 12 hours	3.1±0.8	1.63±0.49	<0.001*
Post operative 16 hours	3.03±0.76	1.67±0.47	<0.001*
Post operative 20 hours	3.13±0.77	1.57±0.504	<0.001*
Post operative 24 hours	3.2±0.82	1.67±0.47	<0.001*

Ramsey sedation score

The mean RSS was higher among patients in Group G compared to Group C as seen in Table 4. The difference in the groups was statistically highly significant when independent ‘t’ test was applied (p<0.001).

**Table 4:** Ramsay sedation scores (RSS) in the two groups at two hours postoperatively. Data presented as Mean±SD. (*) denote p-values with statistically significant differences between the two groups, where p-values are less than 0.05.

RSS	GROUP C	GROUP G	P value
Mean±SD	1.03±0.18	1.73±0.69	<0.001*

Postoperative pain control

The average total dose of analgesics needed in Group C was greater than that in Group G as seen in Table 5. Patients in Group G had a longer mean time to the first rescue analgesia compared to those in Group C as seen in Table 5. The difference in the groups was statistically highly significant when independent ‘t’ test was applied (p<0.001).

**Table 5:** Total dose of analgesics (1=50 mg tramadol) and time for first rescue analgesia in the two groups at different intervals of time (hours). Data presented as Mean±SD. (*) denote p-values with statistically significant differences between the two groups, where p-values are less than 0.05.

	GROUP C	GROUP G	P value
Total doses of analgesic in 24 hours	2±0.12	0.93±0.25	<0.001*
Time for first rescue analgesia (in hours)	9.27±1.7	16.8±3.9	<0.001*

Postoperative nausea and vomiting

About 26.6% of the patients in Group C and 6.7% of the patients in Group G presented with postoperative nausea and vomiting (PONV). Patients in Group C had a higher incidence of PONV as compared to Group G as seen in Table 6. The difference in the groups was statistically significant when the chi-square test was applied (p<0.05).

**Table 6:** Postoperative nausea and vomiting (PONV) in the two groups. Data presented as n(%) (*) denote p-values with statistically significant differences between the two groups, where p-values are less than 0.05.

PONV	GROUP C	GROUP G	P value
Yes	8 (26.6%)	2 (6.7%)	0.04*
No	22 (73.3%)	28 (93.3%)	
Total	30 (100%)	30 (100%)	-

## Discussion

Clonidine, an alpha-2 adrenergic agonist, has been extensively studied for its ability to potentiate the effects of local anesthetics and prolong the duration of sensory and motor blockade when administered orally or intrathecally [6]. The suggested mechanisms of action involve blocking the release of substance P in the pain pathway, decreasing neuronal firing, and amplifying the hyperpolarizing effects of local anesthetics. Gabapentin, an anticonvulsant and analgesic agent, has also been studied for its potential to enhance the effects of local anesthetics and reduce postoperative pain. Its suggested mechanisms include binding to the alpha-2 delta subunit of voltage-gated calcium channels, which regulates influx of calcium and reduces the release of excitatory neurotransmitters [7].

The analysis revealed that the duration of motor blockade was comparable between the study groups, with no statistically significant difference. The duration of sensory blockade was also comparable in Group G (296.53±14.76) and Group C (292.86±31.42). A study by Singh et al. [4] revealed that patients in Group RG experienced a longer duration of postoperative analgesia (248.17±19.6 minutes) compared to those in Group RC (217.36±12.3 minutes). There was disparity in the duration as the technique employed and the drug used were different. In a study by Codi et al. [8] it was found that oral clonidine extended the average duration of motor blockade to 160.8±29.88 minutes, compared to 106.6±9.11 minutes in the control group, with a statistically significant difference (P < 0.05).

Visual Analogue Score

The clonidine group had significantly higher VAS scores for pain at rest compared to the gabapentin group in the postoperative period, starting from 150 minutes onwards. This suggests that gabapentin provided better postoperative analgesia than clonidine. Ghafari et al. [9] and Singhal et al. [10] have demonstrated the efficacy of gabapentin in reducing postoperative pain scores, which corroborates our findings. In a study by Das et al. [11] the differences in VAS scores among the two groups (groups C and G) were not significant at two hours. However, Group G had a lower VAS score value than Group C after four, six, eight, and 24 hours of spinal anesthesia.

Time for first rescue analgesia

It was discovered that there was a statistically significant difference in the amount of time needed to first rescue analgesia between the gabapentin (16.8±3.9) and clonidine (9.27±1.7) groups. The study, done by Mohammadi and Seyedi [12], revealed that the gabapentin group exhibited the lowest usage of rescue analgesic (tramadol). In a study by Turan et al. [13], it was reported that patient satisfaction with postoperative pain management at 24 hours was significantly higher in those treated with gabapentin [85.5 (7.5) vs. 66.5 (15); P < 0.001]. Additionally, oral gabapentin (1.2 g/day) used as an adjunct to epidural analgesia was found to reduce both pain levels and the need for additional analgesics. Baghel et al. [14] found that the total duration of postoperative analgesia, defined as the time from spinal analgesia to the first dose of additional analgesic, was 9.02 hours for Group G compared to 14.20 hours for Group C (P < 0.001). This was in contradiction to the results in our study.

Total requirement of analgesic dose

The cumulative analgesic requirement within the initial 24-hour postoperative period was significantly lower in the gabapentin group compared to the clonidine group, indicating superior efficacy of gabapentin in managing postoperative pain. The findings of the study by Das et al. [11] indicated that within the first 24 hours following spinal anesthesia, Group G required fewer rescue analgesic doses on average (mean of 1.84) compared to Group C (mean of 3). Marashi et al. observed that patients in the gabapentin group required less postoperative morphine compared to those in the clonidine group (P = 0.02) [15]. Similarly, Baghel et al. [14] reported that the mean total dose of analgesic (Inj. diclofenac 1 mg/kg) administered within the first 24 hours was 72.5 mg in Group G and 62.5 mg in Group C.

Side effects

The incidence of PONV was significantly lower in the gabapentin group (6.7%) compared to the clonidine group (26.6%). PONV occurred at a rate of 16% in the clonidine group, 10% in the gabapentin group, and 42% in the control group, according to Gayathri et al. [16]. Alayed et al. found similar outcomes in their trial, with nausea being less common in the gabapentin group than in the control group [17].

In summary, while both clonidine and gabapentin had similar effects on hemodynamic parameters and respiratory rate, gabapentin appeared to provide better postoperative analgesia and a lower incidence of PONV compared to clonidine in our study. These findings are generally in line with the existing literature on the use of these premedications in the perioperative setting. This research aims to provide valuable insights that may refine pain management protocols and improve patient-centered outcomes, ultimately promoting enhanced comfort and facilitating a faster recovery following surgery.

Limitations

Pain perception varies among patients due to differences in pain thresholds, potentially influencing the outcomes. The study was limited to ASA I and II patients, which restricts the applicability of the results to those with higher ASA classifications. Additionally, the exclusion of pediatric and elderly patients limits the relevance of the findings to these populations. Therefore, further research is needed to validate the results across a broader patient population.

## Conclusions

Oral gabapentin at a dose of 600 mg demonstrates greater efficacy as a premedication compared to oral clonidine 100 µg in patients undergoing lower abdominal and lower limb surgeries under spinal anesthesia. Gabapentin provided superior postoperative analgesia, lower VAS scores, and a reduced incidence of PONV, while both medications exhibited comparable effects on motor and sensory blockade, hemodynamic stability, and respiratory function. These results support the use of gabapentin as a more effective premedication in this surgical context.

## References

[1] Rodgers A, Walker N, Schug S (2000). Reduction of postoperative mortality and morbidity with epidural or spinal anaesthesia: results from overview of randomised trials. BMJ.

[2] Vaghadia H (1998). Spinal anaesthesia for outpatients: controversies and new techniques. Can J Anaesth.

[3] Hurwitz EE, Simon M, Vinta SR, Zehm CF, Shabot SM, Minhajuddin A, Abouleish AE (2017). Adding examples to the ASA-physical status classification improves correct assignment to patients. Anesthesiology.

[4] Singh I, Gupta K, Agarwal S, Bansal MK, Samad A, Kalra P (2019). Clinical efficacy of oral gabapentin versus clonidine for preemptive analgesia in knee arthroplasty under epidural anesthesia with 0.75% ropivacaine - a comparative study. Indian J Pain.

[5] McCormack HM, Horne DJ, Sheather S (1988). Clinical applications of visual analogue scales: a critical review. Psychol Med.

[6] Butterworth JF 5th, Strichartz GR (1993). The alpha 2-adrenergic agonists clonidine and guanfacine produce tonic and phasic block of conduction in rat sciatic nerve fibers. Anesth Analg.

[7] Taylor CP (2009). Mechanisms of analgesia by gabapentin and pregabalin--calcium channel alpha2-delta [Cavalpha2-delta] ligands. Pain.

[8] Codi RS, Manimekalai K, Salwe KJ (2013). Effect of oral clonidine premedication on the duration of analgesia produced by spinal bupivacaine. Int J Pharm Bio Sci.

[9] Ghafari MH, Akrami M, Nouralishahi B, Sadegh A (2009). Preoperative gabapentin or clonidine decreases postoperative pain and morphine consumption after abdominal hysterectomy. Res J Biol Sci.

[10] Singhal SK, Kaur K, Arora P (2014). Oral clonidine versus gabapentin as premedicant for obtunding hemodynamic response to laryngoscopy and tracheal intubation. Saudi J Anaesth.

[11] Das R, Paul K, Halder PK, Choudhury A, Roy S, Debbarma A (2022). A comparative evaluation of oral clonidine and oral gabapentin as a premedication on postoperative analgesia duration in patients undergoing spinal anesthesia. Muller J Med Sci Res.

[12] Mohammadi SS, Seyedi M (2008). Effects of gabapentin on early postoperative pain, nausea and vomiting in laparoscopic surgery for assisted reproductive technologies. Pak J Biol Sci.

[13] Turan A, Kaya G, Karamanlioglu B, Pamukçu Z, Apfel CC (2006). Effect of oral gabapentin on postoperative epidural analgesia. Br J Anaesth.

[14] Baghel H, Vatsalya T, Tandon R (2016). Comparative study of single dose pre-emptive gabapentin vs clonidine for post operative pain releif in lower limb surgeries under spinal anaesthesia. Int J Contemp Med Res.

[15] Marashi SM, Morabi AA (2012). The effect of pre-operative oral clonidine or gabapentin on post-operative pain intensity, morphine consumption and post-operative nausea and vomiting in patients who undergone thyroidectomy: a double-blind placebo-control study. J Anesth Clin Res.

[16] Gayathri L, Kuppusamy A, Mirunalini G, Mani K (2023). A comparison between the effects of single-dose oral gabapentin and oral clonidine on hemodynamic parameters in laparoscopic surgeries: a randomized controlled trial. Cureus.

[17] Alayed N, Alghanaim N, Tan X, Tulandi T (2014). Preemptive use of gabapentin in abdominal hysterectomy: a systematic review and meta-analysis. Obstet Gynecol.

